# Implementing health insurance for migrants, Thailand

**DOI:** 10.2471/BLT.16.179606

**Published:** 2017-02-01

**Authors:** Viroj Tangcharoensathien, Aye Aye Thwin, Walaiporn Patcharanarumol

**Affiliations:** a International Health Policy Program, Ministry of Public Health, Tivanond Road, Nonthaburi 11000, Thailand.; b Bureau for Global Health, United States Agency for International Development, Washington, United States of America.

## Abstract

**Problem:**

Undocumented migrant workers are generally ineligible for state social security schemes, and either forego needed health services or pay out of pocket.

**Approach:**

In 2001, the Thai Ministry of Public Health introduced a policy on migrant health. Migrant health insurance is a voluntary scheme, funded by an annual premium paid by workers. It enables access to health care at public facilities and reduces catastrophic health expenditures for undocumented migrants and their dependants. A range of migrant-friendly services, including trained community health volunteers, was introduced in the community and workplace. In 2014, the government introduced a multisectoral policy on migrants, coordinated across the interior, labour, public health and immigration ministries.

**Local setting:**

In 2011, around 0.3 million workers, less than 9% of the estimated migrant labour force of 3.5 million, were covered by Thailand’s social security scheme.

**Relevant changes:**

A review of the latest data showed that from April to July 2016, 1 146 979 people (33.7% of the total estimated migrant labourers of 3 400 787) applied, were screened and were enrolled in the migrant health insurance scheme. Health volunteers, recruited from migrant communities and workplaces are appreciated by local communities and are effective in promoting health and increasing uptake of health services by migrants.

**Lessons learnt:**

The capacity of the health ministry to innovate and manage migrant health insurance was a crucial factor enabling expanded health insurance coverage for undocumented migrants. Continued policy support will be needed to increase recruitment to the insurance scheme and to scale-up migrant-friendly services.

## Résumé

**Problème:**

Les travailleurs migrants en situation irrégulière ne peuvent généralement pas bénéficier des régimes nationaux de sécurité sociale et doivent soit renoncer aux services de santé dont ils ont besoin, soit en assumer eux-mêmes les frais.

**Approche:**

En 2001, le ministère thaïlandais de la Santé publique a mis en place une politique axée sur la santé des migrants. L'assurance maladie des migrants relève d'un régime facultatif, financé par une contribution annuelle payée par les travailleurs. Elle permet d'accéder à des soins de santé dans des établissements publics et réduit les dépenses de santé ruineuses pour les sans-papiers et les personnes à leur charge. Divers services adaptés aux besoins des migrants, comprenant notamment la formation d'agents de santé bénévoles communautaires, ont été mis en place au sein des communautés de migrants et sur les lieux de travail. En 2014, le gouvernement a présenté une politique multisectorielle à l'égard des migrants fondée sur la coordination entre les ministères de l'Intérieur, du Travail, de la Santé publique et de l'Immigration.

**Environnement local:**

En 2011, environ 0,3 million de travailleurs, soit moins de 9% du nombre de migrants actifs – estimé à 3,5 millions –, étaient couverts par le régime de sécurité sociale thaïlandais.

**Changements significatifs:**

Un examen des données les plus récentes a révélé qu'entre avril et juillet 2016, 1 146 979 individus (soit 33,7% du nombre total de travailleurs migrants, estimé à 3 400 787) ont introduit une demande d'inscription, ont été sélectionnés et ont été affiliés au régime d'assurance maladie pour les migrants. Les agents sanitaires bénévoles, recrutés au sein des communautés de migrants et sur les lieux de travail, sont appréciés des communautés locales; ils assurent une promotion efficace de la santé et contribuent à accroître le recours des migrants à des services de santé.

**Leçons tirées:**

La capacité du ministère de la Santé à innover et à gérer l'assurance maladie pour les migrants s'est révélée un facteur déterminant qui a permis d'étendre la couverture d'assurance maladie pour les sans-papiers. Un soutien continu devra être apporté à la politique pour accroître le nombre d'affiliations au régime d'assurance maladie et développer les services adaptés aux besoins des migrants.

## Resumen

**Situación:**

En general, los trabajadores emigrantes indocumentados no suelen ser elegibles para planes de seguridad social nacional, por lo que renuncian a obtener servicios sanitarios necesarios o los pagan de su bolsillo.

**Enfoque:**

En 2001, el Ministerio de Salud Pública tailandés presentó una política en cuanto a la salud de los emigrantes. Los seguros médicos para emigrantes son un plan voluntario, financiado por una prima anual abonada por los trabajadores. Permite el acceso a atención sanitaria en centros públicos y reduce unos gastos sanitarios catastróficos para emigrantes indocumentados y las personas que dependen de ellos. Se presentó una gama de servicios favorables para emigrantes (incluidos voluntarios sanitarios con formación) en la comunidad y en el lugar de trabajo. En 2014, el gobierno presentó una política multisectorial para los emigrantes, coordinada entre los ministerios del Interior, de Trabajo, de Salud Pública y de Inmigración.

**Marco regional:**

En 2011, alrededor de 0,3 millones de trabajadores (menos del 9% de la mano de obra emigrante estimada de 3,5 millones) estaban cubiertos por el plan de seguridad social tailandés.

**Cambios importantes:**

Una revisión de los datos más actualizados mostró que, entre abril y julio de 2016, 1 146 979 personas (el 33,7% del total estimado de trabajadores emigrantes, que suman 3 400 787) solicitaron obtener un seguro médico para emigrantes, fueron examinados e incluidos en el programa. Los voluntarios sanitarios, provenientes de comunidades y lugares de trabajo de emigrantes, son valorados en las comunidades locales y realizan un trabajo eficaz para fomentar la sanidad y aumentar la aceptación de los servicios sanitarios por parte de los emigrantes.

**Lecciones aprendidas:**

La capacidad del Ministerio de Salud para innovar y gestionar los seguros médicos para emigrantes fue un factor crucial que ha permitido la ampliación de la cobertura de los seguros médicos para emigrantes indocumentados. Será necesario un apoyo político continuo para aumentar la captación al plan de seguros y para ampliar los servicios favorables para emigrantes.

## ملخص

**المشكلة:**

لا يكون العاملون من المهاجرين غير الموثقين مؤهلين بشكلٍ عام لبرامج التأمين الاجتماعي الحكومية، مما يضطرهم إما للتنازل عن الخدمات الصحية المطلوبة أو تحمل تكلفتها من مالهم الخاص.

**الأسلوب:**

قامت وزارة الصحة العامة التايلاندية في عام 2001 بطرح سياسة تتعلق بصحة المهاجرين. يعد التأمين الصحي للمهاجرين برنامجًا طوعيًا، يتم تمويله من قبل أقساط التأمين التي يدفعها العاملين سنويًا. ويعمل على تيسير سبل الوصول إلى الرعاية الصحية في المرافق العامة، والحد من النفقات الصحية الضخمة للمهاجرين غير الموثقين وعائلاتهم. وتم تقديم نطاق من الخدمات الرفيقة للمهاجرين، بما في ذلك متطوعين الصحة المجتمعية، في المجتمع ومكان العمل. وفي عام 2014، طرحت الحكومة سياسية تغطي قطاعات عدّة بشأن المهاجرين، والتي تم التنسيق بشأنها عبر وزارات الداخلية والعمل والصحة العامة وشؤون المهاجرين.

**المواقع المحلية:**

في سنة 2011، تم تضمين حوالي 0.3 مليون عامل ­– أي أقل من 9‏% من القوة العاملة المهاجرة المقدرة بـ 3.5 مليون – في برنامج التأمين الاجتماعي بتايلاند.

**التغيّرات ذات الصلة:**

أوضحت مراجعة لأحدث البيانات أنه من نيسان/أبريل وتموز/يوليو 2016، قام 1146979 شخص (33.8% من إجمالي العاملين المهاجرين المقدرين بـ 3400787) بالتقديم في التأمين الصحي للمهاجرين، وتم إخضاعهم للفحص وتسجيلهم في برنامج التأمين الصحي للمهاجرين. ويتم تقدير المتطوعين الصحيين المعينين من المجتمعات وأماكن العمل للمهاجرين من قبل المجتمعات المحلية، ولهم تأثير في تعزيز الصحة وزيادة فرص توصيل الخدمات الصحية للمهاجرين.

**الدروس المستفادة:**

إن قدرة وزارة الصحة على تحسين التأمين الصحي للمهاجرين وإداراته عامل حاسم في تمكين توسيع تغطية التأمين الصحي للمهاجرين غير الموثقين. ويلزم دعم السياسة المستمرة لزيادة التطويع في برنامج التأمين وزيادة الخدمات المناسبة للمهاجرين.

## 摘要

**问题:**

无证移民劳工一般无资格享受国家社会保障计划。他们要么放弃必需的医疗服务，要么自付医疗费用。

**方法:**

2001 年，泰国公共卫生部针对移民医疗问题推出了一项政策。 移民医疗保险计划遵循自愿原则，通过工人每年支付的年度保险金筹措资金。 该计划使得无证移民及其亲属能够在公共机构接受医疗护理，同时减少灾难性医疗费用。 在社区和工作场所推出了多种惠及移民的服务，包括经过培训的社区医疗志愿者。2014 年，政府在内务部、劳工部、公共卫生部和移民部推出了一项针对移民的多部门协调政策。

**当地状况:**

2011 年，加入泰国社会保障计划的移民劳工大约为 30 万人。据估计，在 350 万移民劳工中，参保人数低于 9%。

**相关变化:**

最新的数据显示，从 2016 年 4 月到 7 月，1,146,979 人（据估计，占移民劳工总数 3,400,787 的 33.7%）在提交申请后通过筛选参加了移民医疗保险计划。 从移民社区和工作场所招募的医疗志愿者在当地社区颇受赞赏，这些志愿者有效地改善移民健康状况并且促进移民对医疗服务的理解。

**经验教训:**

卫生部创新和管理移民医疗保险的能力是实现扩大无证移民医疗保险覆盖面的关键因素。 今后还需要继续提供政策支持，增加保险计划人员招募以及提升惠及移民的服务。

## Резюме

**Проблема:**

Незарегистрированные рабочие-мигранты, как правило, не отвечают требованиям для участия в государственных системах социального обеспечения и либо отказываются от получения медицинских услуг, либо оплачивают их за свой счет.

**Подход:**

В 2001 году Министерство здравоохранения Таиланда внедрило стратегическую программу в отношении здравоохранения мигрантов. Медицинское страхование мигрантов является добровольным и финансируется за счет ежегодных страховых взносов, выплачиваемых трудящимися. Такая система позволяет незарегистрированным мигрантам и их иждивенцам получать медико-санитарную помощь в общественных учреждениях и сократить катастрофические расходы на медицинское обслуживание. В общинах и на местах работы был внедрен спектр услуг, ориентированных на мигрантов, в том числе оказываемых подготовленными общинными медицинскими работниками-добровольцами. В 2014 году правительство внедрило многосекторальную политику по мигрантам, скоординированную с министерствами внутренних дел, труда, здравоохранения и иммиграции.

**Местные условия:**

В 2011 году около 0,3 млн рабочих, т. е. менее 9% от подсчитанного количества трудящихся-мигрантов в 3,5 млн человек, участвовали в системе социального обеспечения Таиланда.

**Осуществленные перемены:**

Анализ новейших данных показал, что с апреля по июль 2016 года 1 146 979 человек (33,7% от подсчитанного количества всех мигрантов в 3 400 787 человек) подали заявление на участие, прошли скрининговое обследование и были включены в схему медицинского страхования для мигрантов. Медицинские работники-волонтеры, набранные из общин мигрантов и на местах их работы, высоко ценятся в местных сообществах и эффективно содействуют укреплению здоровья и распространению использования медицинских услуг мигрантами.

**Выводы:**

Потенциал Министерства здравоохранения совершенствования системы медицинского страхования мигрантов и управления ею стал решающим фактором, позволившим распространить охват медицинского страхования на незарегистрированных мигрантов. Постоянная поддержка политики будет необходима для дальнейшего привлечения кадров в систему страхования и для расширения услуг, ориентированных на мигрантов.

## Introduction

Migrants, especially those who are unskilled and undocumented, often work under limited social protection, with poor access to health and other social services, and at risk of exploitation. The International Convention on the Protection of the Rights of All Migrant Workers and the Members of Their Families^1^ was adopted by the United Nations (UN) General Assembly in 1990. However, as of 2016, it was ratified by only 48 UN Member States, most of whom are source countries of international migration. Article 25 of the Convention – which indicates that migrant workers shall enjoy treatment (health) not less favourable than that which applies to nationals – has yet to be fully implemented by States parties to the Convention.

Many countries worldwide face difficulties in meeting the health service needs of migrant workers. Despite regulations on minimum wage and employment benefits, legislation is often not enforced effectively, thus compounding migrants’ vulnerable status. France’s efforts in 1999 to regularize undocumented migrants, by providing limited residence permits and access to state medical aid through means testing, have failed because subsidies were judged to be unaffordable by the government.^2^ The 2012 debt crisis in Europe and the subsequent austerity measures have had a negative impact on social protection for national and non-national labourers.^3^ In Thailand, the growing cost of subsidizing migrant workers’ health care, through exemption of user fees on a humanitarian basis, prompted the government to initiate a health insurance scheme for migrant workers.

## Local setting

According to the most recent figures, the proportion of migrant workers in the total labour force in Thailand grew from 2.2% (0.8 of 34 million) in 1995 to 5.0% (1.8 of 36 million) in 2005.^4^ Mostly coming from Myanmar, migrants are employed in the agriculture and fisheries, construction, manufacturing and service industries, often earning piece-rate wages. Although there are no accurate statistics, around three quarters are estimated to be undocumented migrants (i.e. non-nationals who enter and stay in a country without appropriate legal documentation or, after legally entering, stay beyond the authorized time).^5^ Migrant labour contributed an estimated 6.2% of the Thai gross domestic product of 189.3 billion United States dollars (US$) in 2005, yet the migrant workforce, in particular undocumented workers, were not eligible for tax-supported social benefits.

Migrants who have work permits are fully covered by the Thai social security scheme. This is a mandatory scheme financed by payroll taxes, to which employers, employees and the government contribute equal parts. Thai nationals and migrants who contribute to the social security system have equal rights of access to social security benefits, including health services. In 2011, around 0.3 million workers, less than 9% of Thailand’s estimated migrant labour force of 3.5 million, were covered by the social health insurance scheme. According to the law, a self-employed worker can voluntarily join the social security scheme. However, migrants without legal documents face barriers to enrolling in social health insurance and therefore must resort to paying for health services out of pocket. Undocumented migrants were sometimes exempted from health-care charges, subsidized by hospital revenue, but only at the discretion of hospital staff.

## Approach

Two strands of policy action on migrant health have been introduced in Thailand: (i) extending financial risk protection for migrants and (ii) strengthening the provision of migrant-friendly services.

### Financial risk protection

In 2001 the Thai Ministry of Public Health set up the migrant health insurance scheme for all migrants (documented and undocumented) who are not covered by social health insurance. This was later extended to migrants’ dependants including spouses and children in 2005. Migrant health insurance is a voluntary prepayment scheme financed by an annual premium paid by the migrant worker (2200 baht in 2015, equivalent to US$ 73), with no employer or state contribution, as it is not technically feasible to enforce mandatory participation. 

The scheme has two policy goals: screening for and treatment of certain communicable diseases; and enabling access to health care for migrants. Applying for migrant health insurance requires the migrant to register at a specific hospital where they receive health screening (costing 500 baht in 2005). The screening includes chest X-ray and sputum confirmation for tuberculosis, and tests for syphilis, microfilaria, malaria and leprosy, for which a full course of treatment is offered. The benefit package covers comprehensive curative services, including antiretroviral therapy, and a range of prevention and health promotion services, similar to the Thai universal health coverage scheme. The migrant health insurance excludes some services, such as aesthetic surgery and renal replacement therapy. A full schedule of immunization is provided to child dependants of all migrants. 

### Migrant-friendly services

A second strand of policy action on migrant health was the establishment by the public health ministry in 2003 of innovative, migrant-friendly services with the aim of improving access to health care for all migrants, whether covered by insurance or not. These included the use of volunteer community health workers, mobile clinics for migrant communities, bilingual (mostly Thai and Burmese) signposts and information in health facilities, and outreach services in the workplace.^6^

A pilot programme in seven provinces with large numbers of migrants was set up in 2003 by the public health ministry and the International Organization for Migration with support from the United States Agency for International Development. Volunteer health educators were recruited from migrant communities and workplaces to provide health education and advice about how to access health services. One volunteer for every 50 households was proposed and approved by migrant communities. In manufacturing areas, there were 5–10 volunteers per factory. They received an initial two days’ training by staff at district hospitals, with refresher trainings twice a year.^7^ The training content included personal hygiene, maternal and child health and safe water and sanitation. 

## Relevant changes

Opening up the migrant health insurance scheme to all documented and undocumented migrants and their dependants led to greatly increased enrolment. In the most recent data, between April and July 2016, a total 1 147 889 migrants applied to enrol in the scheme and were screened (33.7% of the total migrant health insurance estimate target of 3 400 787).^8^ Of these, 1 146 979 people were enrolled; 8913 (0.8%) were treated for infectious diseases (4929 of them for pulmonary tuberculosis). A further 910 migrants (0.1%) were assessed as not fit for work (due to substance abuse, syphilis stage 3, symptomatic leprosy or lymphatic filariasis) and returned to their country of origin without treatment (Table 1). All children, not only members of the migrant health insurance scheme, were eligible for full immunization coverage through the same schedule for the expanded programme on immunization as Thai nationals. By 2015, migrant health insurance covered 1.3 million members, including 50 000 children who are targets of immunization. Despite this achievement, the health-service utilization rate among health insurance members was low.^8^

**Table 1:** Outcomes of health screening at registration to the migrant health insurance scheme in Thailand, 1 April 2016 to 2 August 2016

Outcome	No. (%) of migrants
**Migrant health insurance target for 2016**	3 400 787 (100.0)
**Applied for registration and health screening (4 months in 2016)**	1 147 889 (33.8)
**Screened and enrolled into migrant health insurance**	1 146 979 (33.7)
No treatment needed	1 138 066
Treated for infectious diseases	8 913
Pulmonary tuberculosis	4 929
Syphilis	1 913
Lymphatic filariasis	50
Leprosy	5
Other (e.g. such as helminthiasis, anaemia, malnourishment)	2016
**Screened and found not fit for work: returned back to country of origin^a^**	910 (0.1)

^a^ Due to substance abuse, alcoholism, syphilis (stage 3), symptomatic leprosy and lymphatic filariasis.

Source: Health Insurance Group, Ministry of Public Health.^8^^,^^9^

A study in 2008 assessed the migrant health volunteer programme in two provinces having high concentrations of migrant workers.^7^ Interviews with 260 volunteers and 446 migrants showed that community attitudes towards the programme were positive, and the migrants recognized the benefit of these volunteers who spoke the same dialect and shared the same culture. Training of and support to volunteer health communicators had been effective in promoting migrants’ health awareness and improving service uptake through advice and help with navigating through services.^7^ The model was expanded to cover 27 districts in seven provinces with high concentration of migrants, but due to lack of policy support it has yet to be scaled up further.

**Fig. 1:**
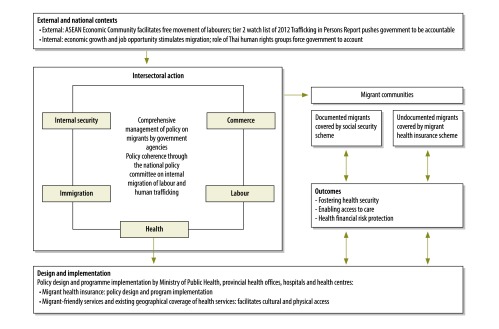
Conceptual framework of comprehensive management for health of migrants in Thailand

Alongside these initiatives were external pressures on the government to take further multisectoral policy on migrants (Fig. 1). When Thailand was listed in the tier 2 watch list of the 2012 Trafficking in Persons Report,^10^ international pressure pushed the government to introduce new initiatives to combat human trafficking.^11^ The Cabinet Resolution on 15 January 2013 endorsed the Ministry of Public Health as the lead agency in providing comprehensive health insurance and service provision to migrant workers and their dependants not covered by the social health insurance. In 2014, a multisectoral policy was introduced and managed by the immigration bureaux of the interior, commerce, labour and public health ministries, encouraging illegal workers to register for temporary permission to stay so that all migrants are screened and covered by health insurance. A national policy committee on international migration of labour and human trafficking was appointed. 

ASEAN: Association of South-East Asian Nations.

## Lessons learnt

Migrant health insurance for undocumented workers in Thailand contributes to health security through screening and treatment of communicable diseases, improved access to health services and reduced risk of catastrophic out-of-pocket expenditure for this vulnerable group. The capacity of the health ministry to innovate and manage migrant health insurance was a crucial factor enabling expanded health insurance coverage for migrants (Box 1).

Box 1 **Summary of main lessons learnt**

- The capacity of the public health ministry to innovate and manage migrant health insurance was a crucial factor enabling expanded health insurance coverage for undocumented migrants.
- Continued policy support will be needed to increase recruitment to the migrant health insurance scheme and to scale-up migrant-friendly services.
- External political pressure can push governments to take action in support of better health care for migrant populations.

Migrant health volunteers acting as communicators in migrant communities and workplaces are important for supporting the scheme by linking with primary health-care facilities and encouraging health-care uptake by migrants. Scaling up migrant-friendly services and recruitment of volunteers will require continued policy support.

Political pressure from outside the country pushed the government to take action in support of better health care for migrant populations. Recognition of labour shortages in some sectors, and migrants’ contribution to the Thai economy, contributed to better inter-sectoral action among the labour, social welfare and health ministries to address these challenges in a holistic way, focusing not only on health concerns (Fig. 1).

Challenges remain, however. Migrants’ experiences of poorly responsive services and fear of litigation by the authorities result in low utilization rates for outpatient and inpatient services.^12^ Health ministry hospitals have a dual role as insurer and provider, and linking the scheme members to a single provider for the whole year is problematic when migrants change employers or move to another province. Portability of insurance coverage has not yet been developed. The voluntary nature of migrant health insurance encourages sick members to participate and healthy persons to self-exclude. Migrants’ illegal status is another key barrier to enrolment. The low enrolment of migrants to migrant health insurance, with limited population coverage, inhibits large pooling of risks, which adversely affects the financial viability of the scheme.
